# Evidence for the placenta-brain axis: multi-omic kernel aggregation predicts intellectual and social impairment in children born extremely preterm

**DOI:** 10.1186/s13229-020-00402-w

**Published:** 2020-12-11

**Authors:** Hudson P. Santos Jr, Arjun Bhattacharya, Robert M. Joseph, Lisa Smeester, Karl C. K. Kuban, Carmen J. Marsit, T. Michael O’Shea, Rebecca C. Fry

**Affiliations:** 1 Biobehavioral Laboratory, School of Nursing, University of North Carolina, 544 Carrington Hall, Campus Box 7460, Chapel Hill, NC 27599-7460 USA; 2 Institute for Environmental Health Solutions, Gillings School of Global Public Health, University of North Carolina, Chapel Hill, NC USA; 3 Department of Pathology and Laboratory Medicine, David Geffen School of Medicine, University of California-Los Angeles, Los Angeles, CA USA; 4 Department of Anatomy and Neurobiology, Boston University School of Medicine, Boston, MA USA; 5 Curriculum in Toxicology and Environmental Medicine, University of North Carolina, Chapel Hill, NC USA; 6 Department of Environmental Sciences and Engineering, Gillings School of Global Public Health, University of North Carolina, Chapel Hill, NC USA; 7 Department of Pediatrics, Division of Pediatric Neurology, Boston University Medical Center, Boston, MA USA; 8 Department of Environmental Health, Emory University, Atlanta, GA 30322 USA; 9 Department of Pediatrics, School of Medicine, University of North Carolina, Chapel Hill, NC USA

**Keywords:** Prenatal neurodevelopmental programming, Social and cognitive impairment, Placental gene regulation, Epigenome-wide association, Differential expression analysis, Multi-omic aggregation

## Abstract

**Background:**

Children born extremely preterm are at heightened risk for intellectual and social impairment, including Autism Spectrum Disorder (ASD). There is increasing evidence for a key role of the placenta in prenatal developmental programming, suggesting that the placenta may, in part, contribute to origins of neurodevelopmental outcomes.

**Methods:**

We examined associations between placental transcriptomic and epigenomic profiles and assessed their ability to predict intellectual and social impairment at age 10 years in 379 children from the Extremely Low Gestational Age Newborn (ELGAN) cohort. Assessment of intellectual ability (IQ) and social function was completed with the Differential Ability Scales-II and Social Responsiveness Scale (SRS), respectively. Examining IQ and SRS allows for studying ASD risk beyond the diagnostic criteria, as IQ and SRS are continuous measures strongly correlated with ASD. Genome-wide mRNA, CpG methylation and miRNA were assayeds with the Illumina Hiseq 2500, HTG EdgeSeq miRNA Whole Transcriptome Assay, and Illumina EPIC/850 K array, respectively. We conducted genome-wide differential analyses of placental mRNA, miRNA, and CpG methylation data. These molecular features were then integrated for a predictive analysis of IQ and SRS outcomes using kernel aggregation regression. We lastly examined associations between ASD and the multi-omic-predicted component of IQ and SRS.

**Results:**

Genes with important roles in neurodevelopment and placental tissue organization were associated with intellectual and social impairment. Kernel aggregations of placental multi-omics strongly predicted intellectual and social function, explaining approximately 8% and 12% of variance in SRS and IQ scores via cross-validation, respectively. Predicted in-sample SRS and IQ showed significant positive and negative associations with ASD case–control status.

**Limitations:**

The ELGAN cohort comprises children born pre-term, and generalization may be affected by unmeasured confounders associated with low gestational age. We conducted external validation of predictive models, though the sample size (N = 49) and the scope of the available out-sample placental dataset are limited. Further validation of the models is merited.

**Conclusions:**

Aggregating information from biomarkers within and among molecular data types improves prediction of complex traits like social and intellectual ability in children born extremely preterm, suggesting that traits within the placenta-brain axis may be omnigenic.

## Background

Despite substantial research efforts to elucidate the etiology of neurodevelopmental impairment [1], little is known about transcriptomic and epigenomic factors influencing trajectories of neurodevelopment, such as those associated with preterm delivery [2]. Children born extremely preterm are at increased risk not only for intellectual impairment but also for Autism Spectrum Disorder (ASD) [3, 4], often accompanied by intellectual disability. In addition, preterm-born children have consistently been observed to manifest social difficulties (e.g., fewer prosocial behaviors) in childhood and adolecense that do not meet diagnostic criteria for ASD [5].

The placenta is posited as a critical determinant of both immediate and long-lasting neurodevelopmental outcomes in children [1]. The placenta is involved in hormone and neurotransmitter production and transfer of nutrients to the fetus, thus having direct influence on brain development. This intimate connection between the placenta and the brain is termed the placenta-brain axis [6, 7]. Epidemiological and animal studies have linked genomic and epigenomic alterations in the placenta with neurodevelopmental disorders and normal neurobehavioral development [8–10]. For example, the Markers of Autism Risk in Babies: Learning Early Signs (MARBLES) study has identified a differentially methylated region containing a putative fetal brain enhancer in placentas from children diagnosed with ASD (*N* = 24) compared to placentas from typically developing (*N* = 23) children [11]. The study of molecular interactions within and between the transcriptome and epigenome that represent the placenta-brain axis may advance our understanding of fetal mechanisms involved in aberrant neurodevelopment [6].

Most prior studies have investigated single molecular levels of the placenta transcriptome or epigenome, precluding analysis of possible interactions that could be linked to neurodevelopmental outcomes. Examining only a single molecular feature, or a single type of feature such as genotype even at a genome-wide scale can still result in much unexplained variation in phenotype due to potentially important interactions between multiple features [12, 13]. This observation is in line with Boyle et al.’s omnigenic model [14, 15], which proposes that gene regulatory networks are so highly interconnected that a large portion of the heritability of complex traits can be explained by effects on genes outside core pathways. Molecular integration to identify placental pathways related to fetal neurodevelopment in children has been largely unexplored but may prove to be insightful in associations with complex diseases [16].

We conducted a genome-wide analysis of DNA methylation (i.e., 5-methylcytosine), miRNA, and mRNA expression in the placenta, examining individual associations with social and intellectual impairment at 10 years of age in children from the Extremely Low Gestational Age Newborn (ELGAN) study [17]. We then combined the transcriptomic and epigenomic data to identify correlative networks of placental biomarkers predictive of social and intellectual impairment as continuous scales, thus allowing us to study neurodevelopmental difficulties beyond the ASD diagnostic categories [18]. To assess the convergent validity of our behavioral findings, we also examined the association of social and intellectual impairment in relation to ASD diagnoses [19]. This is among the first study of its kind to use multiple placental molecular signatures to predict intellectual and social impairment, which may inform a framework for predicting risk of adverse neurocognitive and neurobehavioral outcomes in young children.

## Methods

### ELGAN recruitment and study participants

From 2002 to 2004, women who gave birth at under 28 weeks gestation at one of 14 medical centers across five U.S. states enrolled in the ELGAN study [17]. The Institutional Review Board at each participating institution approved study procedures. Included were 379 of 889 children with both placental molecular data (CpG methylation, mRNA expression, and miRNA expression) and a 10-year neurodevelopment assessment.

### Social and cognitive function and ASD at 10 years of age

Trained child psychologist examiners [5, 20] evaluated general cognitive ability (IQ) with the School-Age Differential Ability Scales-II (DAS-II) Verbal and Nonverbal Reasoning subscales [21]. The Social Responsiveness Scale (SRS) was used to assess severity of ASD-related social deficits in 5 subdomains: social awareness, social cognition, social communication, social motivation, and autistic mannerisms [22]. We used the gender-normed T-score (SRS-T; intended to correct gender differences observed in normative samples) as continuous measure of social deficit [23]. All participants were assessed for ASD [19]. Diagnostic assessment of ASD was conducted with three well-validated measures, administered sequentially. First, the Social Communication Questionnaire (SCQ) was administered to screen for potential ASD, using a score ≥ 11 to increase sensitivity relative to the standard criterion score of ≥ 15 [19, 24]. For children who screened positive on the SCQ criterion, we conducted the Autism Diagnostic Interview–Revised (ADI-R) with the primary caregiver [25]. All children who met ADI-R criteria for ASD, or who had a prior clinical diagnosis of ASD and/or exhibited symptoms of ASD during cognitive testing according to the site psychologist) were then assessed with the Autism Diagnostic Observation Schedule, Second Version (ADOS-2), which served as the criterion measure of ASD in this study [26]. All ADOS-2 administrations were independently scored by a second rater with autism diagnostic and ADOS-2 expertise. In cases of scoring disagreements, consensus was reached via discussion between raters. Item-by-item inter-rater agreement for the 14 ADOS-2 diagnostic algorithm scores was on average 0.93 (SD = 0.12). These developmental assessment procedures and all relevant test scores for ASD and intellectual function are reported in a prior publication [20].

### Placental DNA and RNA extraction

After delivery, placentas were biopsied under sterile conditions. The ELGAN team collected a piece of the chorion, representing the fetal side of the placenta [27]. More specifically, placentas were placed in a sterilized basin and biopsied by pulling back the amnion to expose the chorion at the midpoint of the longest distance between the cord insertion and edge of the placental disk. A sample from the *fetal side* of the placenta was removed by applying traction to the chorion and underlying trophoblast tissue. The specimen was placed in a cryogenic vial and immersed in liquid nitrogen. Specimens were stored at − 80 °C for approximately 13–15 years until processed. For processing, a 0.2 g subsection of the placental tissue was cut from the frozen biopsy and washed with sterile 1 × phosphate-buffered saline to remove any remaining blood. Samples were homogenized using a lysis buffer, and the homogenate was separated into aliquots. This process was detailed in a prior publication [28]. Nucleic acids were extracted from the homogenate using AllPrep DNA/RNA/miRNA Universal kit (Qiagen, Germany). The quantity and quality of DNA and RNA were analyzed using the NanoDrop 1000 spectrophotometer and its integrity verified by the Agilent 2100 BioAnalyzer. As previously described [29], RNA quality was determined using LabChip (Perkin Elmer) instrument to generate RNA integrity numbers (RIN), which ranged from 1 to 3, and DV200 values, which were in acceptable range for placenta tissue [30].

### Epigenome-wide placental DNA methylation

Extracted DNA sequences were bisulfate-converted using the EZ DNA methylation kit (Zymo Research, Irvine, CA) and followed by quantification using the Infinium MethylationEPIC BeadChip (Illumina, San Diego, CA), which measures CpG loci at a single nucleotide resolution, as previously described [27, 28, 31, 32]. Quality control and normalization were performed resulting in 856,832 CpG probes from downstream analysis, with methylation represented as the average methylation level at a single CpG site (*β* value) [28, 33–35]. DNA methylation data was imported into R for pre-processing using the *minfi* package [33]. Quality control was performed at the sample level, excluding samples that failed and technical duplicates; 411 samples were retained for subsequent analyses. Functional normalization was performed with a preliminary step of normal-exponential out-of band (*noob*) correction method [36] for background subtraction and dye normalization, followed by the typical functional normalization method with the top two principal components of the control matrix [34, 37]. Quality control was performed on individual probes by computing a detection *P* value and excluded 806 (0.09%) probes with non-significant detection (*P* > 0.01) for 5% or more of the samples. A total of 856,832 CpG sites were included in the final analyses. Lastly, the *ComBat* function was used from the *sva* package to adjust for batch effects from sample plate [38]. The data were visualized using density distributions at all processing steps. Each probe measured the average methylation level at a single CpG site. Methylation levels were calculated and expressed as *β* values (*β* = intensity of the methylated allele (*M*))/(intensity of the unmethylated allele (*U*) + intensity of the methylated allele (*M*) + 100). *β*values were logit transformed to *M* values for statistical analyses [39].

### Genome-wide placental mRNA and miRNA expression

mRNA expression was determined using the Illumina QuantSeq 3′ mRNA-Seq Library Prep Kit, a method with high strand specificity. mRNA-sequencing libraries were pooled and sequenced (single-end 50 bp) on one lane of the Illumina Hiseq 2500. mRNA were quantified through pseudo-alignment with *Salmon* v.14.0 [40] mapped to the GENCODE Release 31 (GRCh37) reference transcriptome. miRNA expression profiles were assessed using the HTG EdgeSeq miRNA Whole Transcriptome Assay (HTG Molecular Diagnostics, Tucson, AZ). miRNA were aligned to probe sequences and quantified using the HTG EdgeSeq System [41]. Genes and miRNAs with less than 5 counts and variance less than 0.5 for each sample were filtered [42], resulting in 11,224 genes and 2047 miRNAs for downstream analysis. Distributional differences between lanes were first upper-quartile normalized [43]. Unwanted technical and biological variation (e.g. cell-type heterogeneity) was then estimated using *RUVSeq* [44], where we empirically defined transcripts not associated with outcomes of interest as negative control housekeeping probes [45]. One dimension of unwanted variation was removed from the variance-stabilized transformation of the gene expression data using the *limma* package [46, 47].

### Statistical analysis

All code and functions used in the statistical analysis can be found at https://github.com/bhattacharya-a-bt/multiomics_ELGAN.

### Correlative analyses between SRS, IQ, and ASD

Associations among SRS scores, IQ and ASD were assessed using Pearson correlations with estimated 95% confidence intervals, and the difference in distributions of SRS and IQ across ASD case–control was assessed using Wilcoxon rank-sum tests. Associations between demographic variables (race, sex, maternal age, number of gestational days, maternal smoking status, placental inflammation, birth weight* Z*-score and mother’s insurance) with SRS and IQ were determined using multivariable regression, assessing the significance of regression parameters using Wald tests of significance and adjusting for multiple testing with the Benjamini–Hochberg procedure [48].

### Genome-wide molecular associations with SRS and IQ

Once associations between SRS and IQ and ASD were confirmed, we utilized continuous SRS and IQ measures as the main outcomes of interest. Associations between mRNA expression or miRNA expression with SRS and IQ were estimated through a negative binomial linear model using *DESeq2* [46]. Epigenome-wide associations (EWAS) of CpG methylation sites with outcomes were assessed using robust linear regression with test statistic modification through an empirical Bayes procedure [47], described previously [28]. Both the differential mRNA and miRNA expression and EWAS models controlled for the following covariates: race, age, sex, number of gestational age days, birth weight *Z*-score, acute inflammation of the placental chorion, and education level of the mother. As in previous analyses, EWAS models also controlled for five surrogate variables to account for cell-type heterogeneity [28, 38]. Multiple testing was adjusted for using the Benjamini–Hochberg procedure. Sensitivity analyses of test power across various effect sizes (fold change for RNA-seq and miRNA-seq expression) and parameters (mean and dispersion of expression) were conducted for differential gene expression analysis [49], showing sufficient power to detect effect sizes of differential expression (Additional file 2: Supplemental Results—Figure S1A-B). Likewise, we found sufficient power to detect differentially methylated sites at large effect sizes (Additional file 2: Supplemental Results—Figure S1C) using the framework from Mansell et al.[50].

To examine placental cell type variability, we extended the differential mRNA expression analysis to consider cell-type specific proportions. We applied *unmix*, a reference-based deconvolution method [46], to estimate cell-type proportions using reference single cell RNA-seq expression profiles for extravillous trophoblasts, cytotrophoblasts, syncytiotrophoblasts, and mesenchymal stromal cells, derived from fetal placental tissue at 24 weeks of gestation [51]. Here, we refer to the mRNA expression data from ELGAN as the bulk signal, as it represents the mRNA expression from the bulk tissue that includes gene expression signal from all contributing cell (i.e. different trophoblasts, endothelial cells, epithelial cells, etc.). This algorithm estimates the contribution to the bulk mRNA signal from individual cell types on a sample-by-sample basis. We incorporated these cell-type proportions into the differential expression analysis by adding a covariate for cell-type proportions and an interaction term between gene expression and proportion. This cell-type interaction model subtly changes the interpretation of the main gene expression term, representing an estimate of the gene expression effect size on SRS or IQ when the bulk tissue contains 0% of the cell type. Thus, we can detect cell-type-specific differentially expressed genes by testing the interaction effect, which measures how the magnitude of the gene-to-outcome association differs in bulk tissue with 0% and 100% of the cell type in question [52] (details in Additional file 1: Supplemental Methods).

### Placental multi-molecular prediction of SRS and IQ

**Fig. 1:**
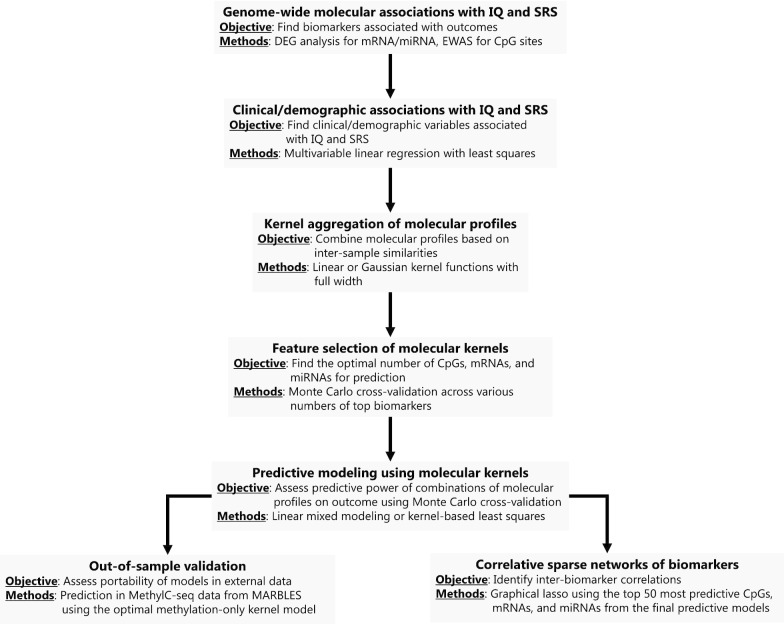
Scheme for kernel aggregation and prediction models. (1) Design matrices for CpG sites, mRNAs, and miRNAs are aggregated to form a linear or Gaussian kernel matrix that measures the similarity of samples. (2) Clinical variables are regressed out of the outcomes IQ and SRS and from the omic kernels to limit influence from these variables. (3) Using 50-fold Monte Carlo cross-validation on 75–25% training-test splits, we train prediction models with the kernel matrices for IQ and SRS in the training set and predict in the test sets. Prediction is assessed in every fold with adjusted ${R}^{2}$ and averaged for an overall prediction metric

We next assessed how well an aggregate of one or more of the molecular datasets (CpG methylation, mRNA expression, and miRNA expression) predicted continuous SRS and IQ scores. The analytical scheme is summarized in Fig. 1, using 379 samples with data for all three molecular datasets (DNA methylation, miRNA, and mRNA). Briefly, we first adjusted the outcome variables and molecular datasets for above noted demographic and clinical covariates using *limma* [53] to account for associations between the outcomes and these coviarates in the eventual predictive models. Next, to model the covariance between samples within a single molecular profile, we aggregated the molecular datasets with thousands of biomarkers each into a *molecular kernel* matrix. A *molecular kernel* matrix represents the inter-sample similarities in a given molecular profile (Additional file 1: Supplemental Methods). A linear or non-linear kernel aggregation may aid in prediction of complex traits by capturing non-additive effects [54–56], which represents a sizable portion of phenotypic variation [57, 58]. Using all individual, pairwise, and triplet-wise combinations of molecular kernel matrices, we fitted predictive models of SRS and IQ based on linear mixed modeling [56] or kernel regression least squares (KRLS) [59] and assessed predictive performance with McNemar’s adjusted *R*^2^ via Monte Carlo cross validation [60]. We also optimized predictive models for the number of included biomarkers per molecular profile with feature selection in the training sets. Extensive model details, as well as alternative models considered, are detailed in Additional file 1: Supplemental Methods.

### Validation in external dataset

Lack of studies that consider placental mRNA, CpG methylation and miRNA data with long-term child neurodevelopment limit the ability to extablish external validation. We obtained one external placental CpG methylation dataset from the MARBLES cohort [11]. To assess out-of-sample performance of kernel models for methylation, we downloaded MethylC-seq data for 47 placenta samples, 24 of which were identified as ASD cases (NCBI Gene Expression Omnibus accession numbers GSE67615) [11]. *β*-values for DNA methylation were extracted from BED files and transformed into *M*-values with an offset of 1 [39], and used the best methylation-only predictive model to predict SRS and IQ in these 47 samples, as detailed in Additional file 1: Supplemental Methods.

### Correlative networks and gene ontology enrichment analysis

In the final KRLS predictive models for both IQ and SRS including all three molecular profiles, we extracted the top 50 most predictive (largest point-wise effect sizes) CpGs, miRNAs, and mRNAs of SRS and IQ. A sparse correlative network was inferred among these biomarkers that links them based on the strength of correlative signals using graphical lasso in *qgraph* [61, 62]. We then conducted biological process and molecular function gene ontology over-representation analysis of genes identified in these correlative networks using WebGestalt [63].

## Results

### Social impairment (SRS) and cognition (IQ) are associated with ASD

**Fig. 2:**
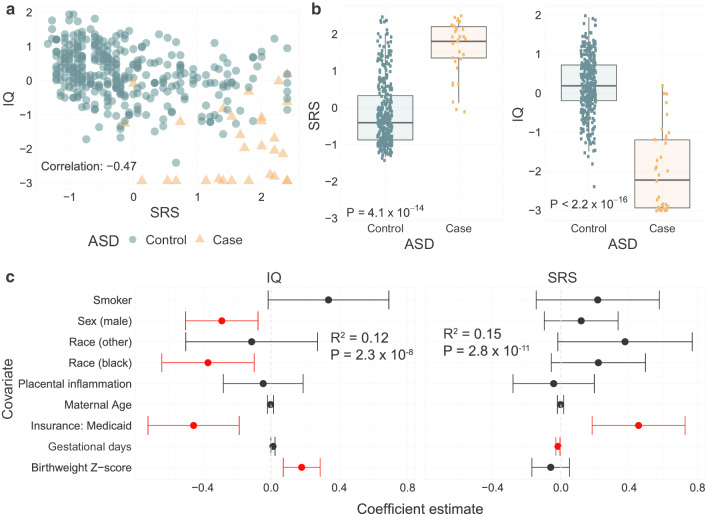
Associations between SRS, IQ, and ASD and with clinical variables. **a** Scatter plot of SRS (X-axis) and IQ (Y-axis) colored by ASD case (orange) and control (blue) status. **b** Boxplots of SRS and IQ across ASD case–control status. *P* value from a two-sample Mann–Whitney test is provided. **c** Caterpillar plot of multivariable linear regression parameters of IQ and SRS using clinical variables. Points give the regression parameter estimates with error bars showing the 95% FDR-adjusted confidence intervals [48]. The null value of 0 is provided for reference with the dotted line

Although the sample is enriched for ASD cases (*N* = 35 cases, 9.3% of the sample) relative to non-preterm cohorts, there is still a relatively low case–control ratio for a genome-wide study of this sample size (descriptive statistics for relevant covariates in Table 1). Therefore, we considered continuous measures of SRS and IQ at age 10 for both associative and predictive analyses. Using continuous variables for SRS and IQ allow us to to study complexities beyond the ASD diagnostic categories [16, 18, 19]. Figure 2a, b shows the relationship between SRS, IQ, and ASD. SRS and IQ are negatively correlated [Pearson *ρ* = − 0.47,  95%CI (− 0.55, − 0.39)]. The mean SRS is significantly higher in ASD cases compared to controls [mean difference of 1.74, 95%CI (1.41, 2.07)]. Mean IQ is significantly lower in ASD cases versus controls [mean difference of − 2.23, 95%CI (− 2.46, − 1.96)]. We also measured associations between demographic characteristics with SRS and IQ using multivariable regression (Fig. 2c). Male sex is associated with lower IQ, while public health insurance is associated with both lower IQ and increased social impairment. Demographic variables included in the multivariable regression explain approximately 12% and 15% of the total variance explained in IQ and SRS, as measured by adjusted *R*^2^, with a summary of regression parameters in Table 2. Based on the associations identified here and the value of inclusion of continuous measures, subsequent transcriptomic and epigenomic analyses control for demographic covariates.

**Table 1:** Descriptive statistics for demographic and clinical covariates

Continuous variable	Mean, SD, median
Maternal age (years)	29.6, 6.61, 29.5
Gestational age (days)	182.5, 9.17, 184.0
Birthweight Z-score	0, 1, 0.05

Categorical variable	Number (proportion)
ASD	
Case	35 (9.3%)
Control	344 (90.7%)
Race	
White	233 (61.5%)
Black	112 (29.5%)
Other	34 (9.0%)
Sex of baby	
Female	180 (47.5%)
Male	199 (52.5%)
Mother’s smoking status	
Non-smoker	340 (89.7%)
Smoker	39 (10.3%)
Mother’s insurance status	
Private	251 (66.2%)
Medicaid	128 (33.8%)
Placental chorion inflammation	
Not inflamed	252 (66.5%)
Inflamed	127 (33.6%)

**Table 2:** Summary of multivariable regression models of SRS and IQ in relation to clinical covariates (self-reported race, sex, maternal age, smoking status, insurance level of the mother, gestational age, birthweight *Z*-score, and inflammation of the placental chorion)

Parameter	SRS		IQ	
	Estimate (SE)	FDR-adjusted *P* value (Raw *P* value)	Estimate (SE)	FDR-adjusted *P* value (Raw *P* value)
Race				
Black	0.219 (0.13)	0.165 (0.091)	− 0.369 (0.13)	0.012 (0.004)
Other	0.375 (0.19)	0.087 (0.043)	− 0.113 (0.18)	0.684 (0.533)
Sex				
Male	0.119 (0.10)	0.342 (0.243)	− 0.288 (0.10)	0.012 (0.004)
Maternal age	− 0.002 (0.01)	0.800 (0.800)	− 0.003 (0.01)	0.792 (0.748)
Smoking status				
Yes	0.215 (0.17)	0.334 (0.204)	0.337 (0.17)	0.087 (0.043)
Mother’s insurance				
Medicaid	0.454 (0.13)	0.002 (0.001)	− 0.453 (0.13)	0.003 (0.001)
Gestational age	− 0.017 (0.01)	0.012 (0.002)	0.012 (0.01)	0.087 (0.043)
Birthweight Z-score	− 0.060 (0.05)	0.342 (0.247)	0.179 (0.05)	0.003 (0.001)
Placental inflammation	− 0.042 (0.11)	0.793 (0.705)	− 0.046 (0.11)	0.793 (0.677)

### Genome-wide associations of mRNA, miRNA, and CpGs with SRS and IQ

Genome-wide association tests between each of the individual placental molecular datasets (e.g. the placental mRNA data, the CpG methylation, or the miRNA datasets) in relation to SRS and IQ (see "Methods") identified two genes with mRNA expression significantly associated with SRS at FDR-adjusted *P* < 0.01, namely Hdc Homolog, Cell Cycle Regulator (*HECA*), and LIM Domain Only 4 (*LMO4*). We did not find CpG sites or miRNAs associated with SRS (Table 3). Associations between IQ and the mRNA expression, at FDR-adjusted *P*  <  0.01, were observed at four genes, namely Ras-Related Protein Rab-5A (*RAB5A*), Transmembrane Protein 167A (*TMEM167A*), Signal Transducer and Activator of Transcription 2 (*STAT2*), ITPRIP Like 2 (*ITPRIPL2*). One CpG site, *cg09418354*, located in the gene Carbohydrate Sulfotransferase 11 (*CHST11*) displayed an association with IQ, and no miRNAs were associated with IQ (Table 3). Manhattan plots (Additional file 2: Supplemental Results—Figure S2) show the strength of associations of all biomarkers by genomic position. No mRNAs, CpG sites, or miRNAs were significantly associated with both SRS and IQ. Summary statistics for these associations are provided in Additional file 2: Supplemental Results: Table S1.

**Table 3:** Summary of genome-wide associations of molecular profiles with SRS and IQ at FDR-adjusted $P < 0.01$

Biomarker	Effect size	FDR-adjusted ${P }$value
*SRS*		
mRNA expression		
*HECA*	0.571	0.001
*LMO4*	0.467	0.001
*IQ*		
Biomarker		
mRNA expression		
*RAB5A*	− 0.516	0.002
*TMEM167A*	− 0.632	0.004
*ITPRIPL2*	− 0.557	0.004
*STAT2*	− 0.584	0.004
CpG methylation site		
cg09418354 (within *CHST11*)	− 0.005	0.002

We also considered differential mRNA expression analysis specific to four key distinct cell-types that comprise the placenta: extravillous trophoblasts, cytotrophobalsts, syncytiotrophoblasts, and mesenchymal stromal cells [64]. Importantly, we did not detect any significant associations between placental cell-type proportions and the differentially expressed genes in the bulk tissue. Incorporating estimated cell-type proportions into an interaction-based differential mRNA expression model revealed no cell-type-specific differentially expressed genes at FDR-adjusted *P* < 0.01. To examine any cell-specific trends, at FDR-adjusted *P* < 0.05, we detected two SRS-associated stromal cell-specific differentially expression genes and two IQ-associated syncytiotrophoblast-specific differentially expression genes (Additional file 2: Supplemental Results—Table S2), all not detected without the interaction model. These SRS-associated genes include Bromodomain Containing 2 (*BRD2*), associated with fetal metabolic programming of newborns [65]. Furthermore, we detected a syncytiotrophoblasts-specific association between IQ and ATPase Plasma Membrane Ca2 + Transporting 1 (*ATP2B1*), a gene whose polymorphic variants have been shown to have associations with preeclampsia [66, 67].

### Kernel regression shows predictive utility in aggregating multiple molecular datasets

**Fig. 3:**
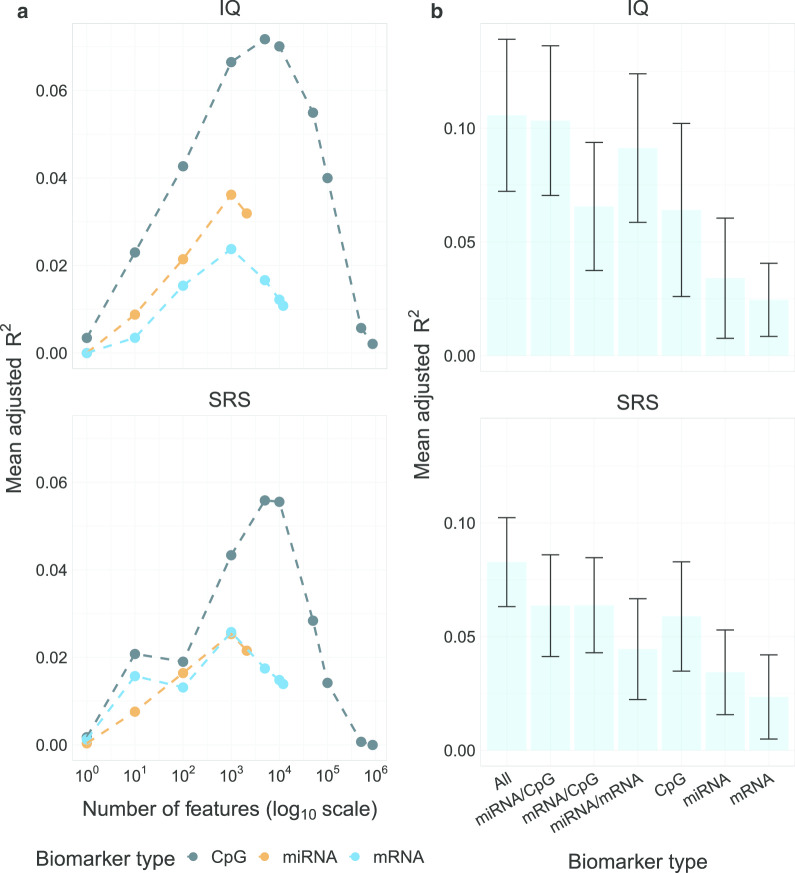
In-sample predictive performance of kernel models. **a** Adjusted mean ${R}^{2}$ (*Y*-axis) of best kernel models over various numbers of the top biomarkers (*X*-axis) in the CpG (dark blue), miRNA (orange), and mRNA (light blue) omics over 50 Monte Carlo folds. The *X*-axis scale is logarithmic. **b** Bar plots of adjusted mean ${R}^{2}$ (*Y*-axis) for optimally tuned kernel predictive models using all combinations of omics (*X*-axis) over 50 Monte Carlo folds. The error bar gives a spread of one standard deviation around the mean adjusted ${R}^{2}$

Because the genome-wide association analyses revealed few mRNAs, CpG sites or miRNAs that were associated with SRS or IQ with large effect sizes, we next assessed the impact of aggregating these molecular datasets on prediction of SRS and IQ. This was done to account for the considerable number of biomarkers that have moderate effect sizes on outcome. To find the most parsimonious model with the greatest predictive performance, we first selected the optimal number of biomarkers per molecular profile from the training set for each outcome that gave the largest mean adjusted *R*^2^ in predictive models with only one of the three molecular datasets (see Additional file 1: Supplemental Methods). Figure 3a shows the relationship between the number of biomarkers from the mRNA expression, CpG level, miRNA expression datasets and their predictive performance. In general, predictive performance steadily increased as the number of biomarker features increased until reaching a tipping point where predictive performance decreased (Fig. 3a). Overall, for CpG methylation, the top (lowest *P* values of association) 5000 CpG features showed the greatest predictive performance, and for the mRNA and miRNA expression datasets, the top 1000 features showed the greatest predictive performance.

Using the fully-tuned 7000 biomarkers (5000 for CpG methylation and 1000 for both mRNA and miRNA expression) per molecular dataset with feature selection carried out in the training set, we trained predictive models (both linear and Gaussian kernel models) using all individual, pair-wise, and triplet-based combinations of the three molecular datasets. Figure 3b shows that whereas the mRNA had the lowest predicted performance to both IQ (*R*^2^ = 0.025) and SRS (*R*^2^ = 0.025), aggregating the mRNA expression, CpG methylation and miRNA expression datasets tends to increase the predictive performance. Specifically, in relation to both outcomes (SRS and IQ), the model using all three integrated datasets shows the greatest predictive performance (mean adjusted *R*^2^ = 0.11 in relation to IQ and *R*^2^ = 0.08 in relation to SRS).

### Correlative networks of placental biomarkers

To gain further understanding of the associations among the identified mRNA, CpG and miRNA biomarkers in the context of IQ and SRS, we extracted (*n* = 50) mRNA, CpGs, and miRNAs with the largest effect sizes on IQ and SRS in the kernel regression models and inferred sparse correlative networks using the graphical lasso [61, 62] (see "Methods"). In the networks (Additional file 2: Supplemental Results—Figure S3), each molecular dataset clusters by itself, with minimal nodes extending between molecular datasets, and more correlation observed between miRNAs and CpG methylation versus mRNAs. These networks point to genes that have been shown in literature to play important roles in neuronal development and in placental function. For example, *SMARCA2* (SWI/SNF Related, Matrix Associated, Actin Dependent Regulator Of Chromatin, Subfamily A, Member 2) and *DDX59* has been implicated in development disorders of the brain, such as Nicolaides-Baraitser syndrome and epilepsy, and other developmental disorders, such as dysfunctional central nervous system development and orofaciodigital syndrome [68–73]. Furthermore, *ARL5B* (ADP Ribosylation Factor Like GTPase 5B) and *MPP5* (Membrane Palmitoylated Protein 5) have been associated with decidua and trophoblasts functions within the placenta [74–76]. Furthermore, at FDR-adjusted *P* < 0.05, over-representation analysis revealed gene enrichments for membrane organization processes (endomembrane system and membrane organization) for the IQ-associated gene set and nucleic acid and enzyme binding processes (RNA binding, ubiquitin protein ligase binding, heterocyclic compound binding, etc.) for the SRS-associated gene set (Additional file 2: Supplemental Results—Table S3).

### Validation of in-sample and out-sample SRS and IQ prediction with ASD case and control

To contextualize our predictions, we tested whether the predicted SRS and IQ scores generated by our kernel models are associated with ASD case–control status; these predicted SRS and IQ scores represent the portion of the observed SRS and IQ values that our models can predict from placental genomic features. We used the optimal 7000 biomarker features identified with a tenfold cross-validation process, splitting samples into 10 hold-out sets and using the remaining samples as a training set to predict SRS and IQ for all 379 samples. After accounting for covariates, the predicted SRS and IQ values from the biomarker data were well-correlated with the observed clinical SRS and IQ values, explaining approximately 8% (approximate Spearman *ρ* =0.29, cross-validation *R*^2^ *P* value *P* = 7.5 × 10^−9^) and 12% (Spearman *ρ* = 0.35, *P* = 3.6 × 10^−12^) of the variance in the observed SRS and IQ variables, respectively. This shows that biomarkers in the placenta can explain considerable amount of the variance in SRS and IQ at 10 years of age.

**Fig. 4:**
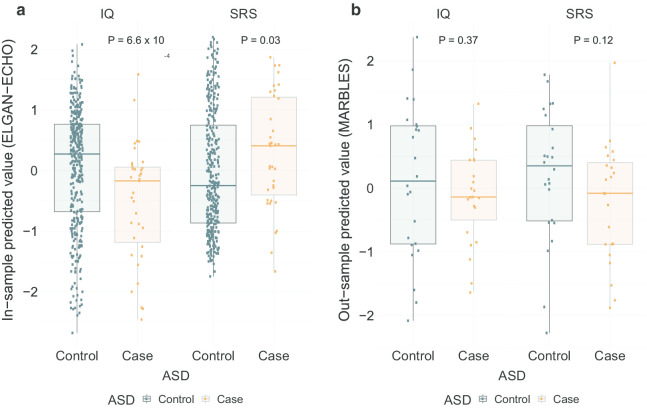
Association of ASD case/control status with predicted SRS and IQ. **a** Box-plots of in-sample predicted IQ (left) and SRS (right) over ASD case/control in ELGAN over tenfold cross-validation. **b** Box-plots of out-sample predicted IQ (left) and SRS (right) over ASD case/control in MARBLES external validation dataset. $P$-values presented as from a Mann–Whitney test of differences across the ASD case/control groups

Lastly, we assessed associations between molecularly-predicted SRS and IQ values and ASD case–control status. In ELGAN, we found strong associations between the predicted SRS and IQ with ASD case and controls, mean difference of − 0.56 (test statistic *W* = 8121, *P* = 6.6 × 10^−4^) for IQ, and mean difference of 0.33 (*W* = 4717, *P* = 0.03) for SRS (Fig. 4a). Because of the lack of an available external dataset with all three molecular data (mRNA, CpG methylation, and miRNA) and IQ, SRS and ASD data, we assessed the out-of-sample predictive performance of the CpG methylation-only models using MethylC-seq data from the MARBLES cohort (GEO GSE67615) [11]. We computed predicted IQ and SRS values for 47 placental samples (24 cases of ASD) and assessed differences in mean predicted IQ and SRS across ASD case and control groups. The direction of the association is similar to our data for IQ, yet the differences in mean-predicted IQ (− 0.22, *P* = 0.37) and SRS (− 0.42, *P* = 0.12) across ASD groups in MARBLES are not significant (Fig. 4b). This external validation provides some evidence of the portability of our models and merits further future validation of these models, as more placental multi-omic datasets are collected.

## Discussion

We evaluated the predictive capability of three types of molecular biomarker data, namely transcriptomic (mRNA), and epigenomic (miRNA expression, CpG methylation), in the placenta on cognitive and social impairment in relation to ASD at 10 years of age. The molecular biomarker data highlight that genes that play important roles in placental functioning (*ARL5B* and *MPP5*) and neurodevelopment (*SMARCA2*, *DDX59, MPP5*) were associated with or predictive of SRS and IQ. The multi-omic predictions of SRS and IQ are strong and explain up to 8% and 12% of the variance in the observed SRS and IQ variables in tenfold cross-validation, respectively. External validation of our models is inconclusive, however, and merits further investigation to minimize uncertainty in our findings, as mentioned in the limitations section. This study supports the utility of aggregating information from biomarkers within and among molecular datasets to improve prediction of complex neurodevelopmental outcomes like social and intellectual ability, suggesting that traits on the placenta-brain axis may be omnigenic.

Several genes with known ties to neurodevelopmental disorders distinguished individuals with and without intellectual or social impairments. For example, *LMO4* (associated with social impairment) is a protein encoding gene with a broad spectrum of expression in human tissues and involved in multiple developmental pathways, including neurogenesis. Among its many roles, *LMO4* promotes the acquisition of cortical neuronal identities by forming a complex with the protein neurogenin 2 (NGN2) and subsequently activating NGN2-dependent gene expression [77]. Humans with deletions in *LMO4* display intellectual disabilities and occasionally autism [78]. Furthermore, this gene has also been associated with modulation of fear [79] and cue-reward leaning [80], which could result in perceptions and behaviors seen in association with social impairment. *LMO4* also influence the growth factor-*β* (TGF-*β*) cytokine pathway, which plays important roles in mammalian development [81]. *LMO4* and *HECA* has been identified in pathways and processes related to neural development via commonly regulated targets of Forkhead box protein P2 (FOXP2) and miR-3666; the *LMO4* gene shows asymmetric expression in the embryonic brain possibly due to repression by FOXP2 and hence plays important roles in cortical patterning [82].

Among the biomarkers associated with IQ, we found *RAB5A*, a protein coding gene belonging to a family of small GTPases, involved in a variety of cellular processes including intracellular membrane trafficking. In the placental syncytiotrophoblasts, a specialized epithelial structure that interfaces the placenta and maternal blood, *RAB5A* has shown involvement in vesicular trafficking which could affect the syncytiotrophoblast function of transporting nutrients necessary for fetal development [83]. *RAB5A* can also affect the regulation of genes with roles in cell proliferation [84]. In terms of cognitive outcomes, *RAB5A* and the RAB family play critical roles in synaptic function [85, 86] and dendritic branching [87]. Finally, genetic variants of *RAB5A* have been associated with ASD [88]. Other relevant genes are *STAT2* and *CHST11*. *STAT2* is a well-known essential and specific positive effector of type I interferons (IFNs) signaling [89], and placental type I IFNs is an important immune modulator, including modulation of viral infection in the mother and fetus [90]. *STAT2* was identified as one differentially expressed genes in ASD and co-morbidities that overlap with innate immunity pathways [91]. Also, with important roles in immune regulation, the genetic variation and methylation of the *CHST11* gene, for which we found a methylated CpG site associated with intellectual impairment, has been linked to neurodevelopmental disorders [92, 93].

In our correlative gene network analysis, we detected a group of inter-related genes associated with IQ enriched for membrane organization processes. This enrichment may point to a previously established link: endothelial cell membrane dysfunction leads to deficient nutrient exchange and has a lasting impact on neurodevelopment [94]. For example, one of these genes is *DDX59*; in our differential expression analysis, *DDX59* shows a nominally significant negative association with IQ. A recent study has shown that *DDX59* is upregulated in syncytiotrophoblasts in severe preeclampsia patients compared to controls [95]. Another example is *MPP5*, expressed in the placenta, brain, nervous system and other tissues, which is essential for cell polarity, fate and survival. In the placenta, *MPP5* seem to have significant roles: promotion of embryo-decidual adhesion [75], differentiation of extravillous trophoblasts [76], and gene expression levels from chorionic villus have been associated with severe early-onset preeclampsia [96]. In terms of neurodevelopment, both animal and human studies show that *MPP5* has been found to be essential in neurogenesis [97, 98]; in a murine model *Mpp5* depletion led to microcephaly, decreased cerebellar volume and cortical thickness, while humans with de novo variants of MPP5 suffer from global developmental delays with language regression and behavioral changes [97]. In our differential expression analysis, we found that *MPP5* has a nominal negative association with IQ. Lastly, we also estimated that *SMARCA2* has a strong predictive effect on and a nominally positive association with IQ; previous literature shows that epigenomic effects on and genetic dysfunction of *SMARCA2* plays a role in development of Nicolaides-Baraitser syndrome, a developmental disorder categorized by intellectual disability and seizures [68, 69]. It is worth noting that these results are ultimately correlational in nature, and a causal interpretation should be avoided. Future research using in vitro and in vivo studies could elucidate the mechanistic influences of placental expression of these genes on the brain.

Not only did our cell-type-specific differential expression analysis show that the differentially expressed genes we detected in the bulk placenta were minimally affected by cell-type heterogeneity, we detected genes whose cell-type-specific expression has large associations with IQ and SRS. For instance, we detected a syncytiotrophoblast-specific association between IQ and *ATP2B1*, a gene that has been implicated in preeclampsia [66, 67], an in utero condition that is partially mediated by dysfunctional syncytiotrophoblasts [99] and has negative impacts with childhood neurodevelopment [100, 101]. In addition, *ATP2B1* encodes PMCA1, a plasma membrane calcium ion pump, shown to have reduced activity in fetal-facing syncytiotrophoblast basal plasma membranes in patients with preeclampsia compared to controls [102–105]. This cell-type-specific analysis underscores the importance of not only accounting for cell-type-heterogeneity in bioinformatics analyses of the placenta but estimating cell-type-specific associations through deconvolution or single cell assays.

Comparing the individual molecular datasets, DNA methylation effects showed the strongest prediction of both SRS and IQ impairment. There is strong evidence suggesting inverse correlation between DNA methylation of the first intron or promoter region and gene expression across tissues and species [106]. We found that many of the CpG loci with the largest effect sizes on SRS and IQ identified in our analysis are located in genes with DNase hyperactivity or active regulatory elements for the placenta [107, 108], suggesting that these loci likely play regulatory functions. Experimental studies have demonstrated regions of the genome in which DNA methylation is causally important for gene regulation and those in which it is effectively silent [109]. We found that aggregating biomarkers within and among molecular datasets improves prediction of social and cognitive impairment. Specifially, this observation suggests new possibilities to the discovery of candidate genes in the placenta that convey neurodevelopmental risk, improving the understanding of the placenta-brain axis. Recent work in transcriptome-wide association studies (TWAS) are a promising tool that aggregates genetics and transcriptomics to identify candidate trait-associated genes [110, 111]. Incorporating information from regulatory biomarkers, like transcription factors and miRNAs, into TWAS increases study power to generate hypotheses about regulation [112, 113]. Given our observations in this analysis and the number of the integrated molecular datasets, we believe that the ELGAN study can be used to train predictive models for placental transcriptomics from genetics, enriched for regulatory elements [113]. These transcriptomic models can then be applied to genome-wide association study cohorts to study the regulation of gene-trait associations in the placenta.

## Limitations

When interpreting the results of this study, some factors should be considered. Extremely preterm birth is strongly associated with increased risk for neurodevelopmental disorders [19]. This association may lead to bias in estimated associations between the molecular biomarkers and outcomes, mainly when unmeasured confounders are linked to both pre-term birth and autism [114]. Ideally, an external dataset with both multiomic data and pre-term birth phenotypes could be used to examine associations between molecular profiles and pre-term birth to investigate the degree to which collider bias affects associations between molecular profiles and SRS and IQ [114, 115]. Without this assessment of collider bias, results from our predictive models may not generalizable to term cohorts. Still, to our knowledge the ELGAN cohort is among the largest available placental repositories with both multiple molecular datasets and long-term neurodevelopmental assessment of the children.

Second, as the placenta is comprised of several heterogeneous cell types, cell type-specific molecular patterns in the placenta should be taken into consideration when interpreting these findings. We did consider deconvolution of our tissue samples using mRNA expression. Due to a dearth of placental cell-type-specific expression references, we opted for a reference-based deconvolution of four key cell types. These four cell types, however, do not fully represent the cellular compexity of the placenta. As these reference expression profiles become available, a more comprehensive analysis with reference-based deconvolution may reveal more cell-type-specific expression and methylation patterns that are specific to diverse populations of trophoblasts, stromal cells, endothelial cells, and pericytes. There are also considerations made about the degradation of RNA in the placental specimens over time. As the placental tissue was stored for ~ 15 years, we had to impose strict pre-filtering of genes whose expression have low counts and dispersions [42], resulting in a reduction of the analyzable transcriptome.

Lastly, to test the reproducibility and robustness of our kernel models, further out-of-sample validation is required, using datasets with larger sample sizes and similar molecular datasets. Though in-sample predictive performance is strong, platform differences between the ELGAN training set (assayed with the EPIC BeadChip) and the validation set (assayed with MethylC-seq) may lead to loss of predictive power. As our optimal models trained in ELGAN all aggregated the DNA methylation, miRNA, and mRNA datasets, the dearth of data for the placenta, in the context of social and intellectual impairment, makes out-of-sample validation of the full model especially challenging. In spite of these limitations, these data support the association between molecular features within the fetal placenta and social and cognitive outcomes in children that merits future investigation.

## Conclusions

Our analysis underscores the importance of synthesizing data representing various levels of biological organization to understand distinct transriptomic and epigenomic underpinnings of complex developmental deficits, like intellectual and social impairment. This study provides novel evidence for the omnigenicity of the placenta-brain axis in the context of social and intellectual impairment.

## Supplementary information

**Additional file 1.** Supplemental methods.

**Additional file 2.** Supplemental results.

## Data Availability

mRNA and miRNA expression data from the ELGAN study is available from the NCBI Gene Expression Omnibus GSE154829. CpG methylation data is freely available and can be requested from H.S.P or R.C.F. For validation, we used MethylC-seq data from the MARBLES study available at GSE67615. Single cell RNA-seq data used for reference-based deconvolution is available at GSE89497. Supplemental code and results are provided on Github: https://github.com/bhattacharya-a-bt/multiomics_ELGAN. Any questions about data availability can be directed to H.P.S.

## Abbreviations

ASD: Autism Spectrum Disorder
DAS-II: Differential Ability Scales-II
ELGAN: Extremely Low Gestational Age Newborn
EWAS: Epigenome-wide association study
IQ: Intellectual ability
KRLS: Kernel regression least squares
MARBLES: Markers of Autism Risk in Babies-Learning Early Signs
SRS: Social Responsiveness Scale
SRS-T: SRS gender-normed T-score
