# Supplementary material for: Evidence for the placenta-brain axis: multi-omic kernel aggregation predicts intellectual and social impairment in children born extremely preterm
Source: Mol Autism. 2020 Dec 11;11:97. doi: 10.1186/s13229-020-00402-w (PMC7730750; doi:10.1186/s13229-020-00402-w)
Supplement: Supplementary file 1 — Additional file 1. Supplemental methods. [file 13229_2020_402_MOESM1_ESM.docx]

**Supplemental Methods**

***Projection to kernel space***

For a given omic design matrix $\boldsymbol{X}_{m}$ with $n$ samples on the rows and $p_{m}$ features on the columns, with each column standard-normalized to zero mean and unit variance, we project the omic into a sparse kernel space using the simple linear kernel:

$${\boldsymbol{K}_{\boldsymbol{linear}}}_{m} = {p_{m}^{-1}\boldsymbol{X}}_{m}\boldsymbol{X}_{m}^{T},$$

resulting in ${\boldsymbol{K}_{\boldsymbol{linear}}}_{m}$, an $n\times n$ matrix. We call ${\boldsymbol{K}_{\boldsymbol{linear}}}_{m}$ the omic linear kernel matrix for omic $m$.

Alternatively, for a given omic $\boldsymbol{X}_{m}$ on $n$ samples (rows) and $p_{m}$ features (columns), with each column standard-normalized to zero mean and unit variance, we project the omic design matrix into a sparse Gaussian kernel space using the Gaussian kernel function. For samples $i$ and $j$, corresponding to the $i$th and $j$th rows of $\boldsymbol{X}_{m}$, the $i,j$-th element of the Gaussian kernel matrix ${\boldsymbol{K}_{\boldsymbol{Gauss}}}_{m}$ for omic $m$ is defined as

${\boldsymbol{K}_{\boldsymbol{Gauss}}}_{m}=\exp\left( \frac{-\left\| x_{i.}-x_{j.} \right\|^{2}}{p_{m}} \right)$.

To compare two omic kernels $\boldsymbol{K}_{m_{1}}$and $\boldsymbol{K}_{m_{2}}$ in the same kernel space (i.e. two linear kernel matrices or two Gaussian kernel matrices), we compute their kernel alignment $S$, a measure of similarity between two omic kernels [1] using the standardized Frobenius inner product of two matrices:

$S= \frac{\left\langle\boldsymbol{K}_{m_{1}},\boldsymbol{K}_{m_{2}} \right\rangle_{F}}{\sqrt{\left\langle\boldsymbol{K}_{m_{1}},\boldsymbol{K}_{m_{1}} \right\rangle_{F}\left\langle\boldsymbol{K}_{m_{2}},\boldsymbol{K}_{m_{2}} \right\rangle_{F}}}$,

where, for two $n\times n$ matrices $\boldsymbol{A}$ and $\boldsymbol{B}$, $\left\langle\boldsymbol{A},\boldsymbol{B} \right\rangle_{F}= \sum_{i,j=1}^{n} A_{ij}B_{ij}$.

***Multi-omic kernel predictive model with linear kernels***

We consider the following linear model, assuming $M$ total omics considered:

$$\boldsymbol{Y}=\boldsymbol{X}_{c}\boldsymbol{\beta}_{C}+\boldsymbol{U}+\boldsymbol{\epsilon},$$

where $\boldsymbol{Y}$ is an outcome of interest (either SRS or IQ), standardized to zero mean and unit variance, $\boldsymbol{X}_{C}$ is the design matrix of covariates, $\boldsymbol{\beta}_{C}$ is a vector of fixed effects for covariates, $\boldsymbol{U}$ is a vector of omic predictive scores, and $\epsilon$ is Gaussian random error with zero mean and identity covariance matrix. We assume that the omic predictive scores are normally distributed with zero mean and a fused kernel matrix as its variance-covariance matrix, i.e. $\boldsymbol{U}\sim N(0,\boldsymbol{K})$ and $\boldsymbol{K}= \sum_{m=1}^{M} \boldsymbol{K}_{m}$. This model is simply a traditional linear mixed model, treating clinical covariates as fixed effects and aggregating all omics into omic predictive scores that are treated as random effects clustered around zero. It is straightforward that

$$\boldsymbol{U}= \sum_{m=1}^{M} \boldsymbol{U}_{m}= \sum_{m=1}^{M} \sum_{j=1}^{p_{m}} \boldsymbol{X}_{mj}\eta_{mj},$$

where $\boldsymbol{U}_{m}$ is the vector of omic predictive scores for the $m$th omic profile and the weight sum of omic features $\boldsymbol{X}_{mj}$ with random effects coefficients $\eta_{mj}$. Assuming $\eta_{mj}\sim N(0,\frac{\sigma_{m}^{2}}{p_{m}})$, we see that $\boldsymbol{U}_{m}\sim N(0,\sigma_{m}^{2}\boldsymbol{K}_{m})$ and $\boldsymbol{U}\sim N\left( 0,\boldsymbol{K} \right).$

***Kernel modeling training and evaluation for linear kernels***

We evaluate our models through 50-fold Monte Carlo cross-validation, using methods similar to Zhu et al [2]. Given a single cross-validation fold, with 75% of the data in training set and 25% in a test set, we can divide $\boldsymbol{Y}=\left[ \begin{matrix} \boldsymbol{Y}_{train} \\ \boldsymbol{Y}_{test} \end{matrix} \right]$ and $\boldsymbol{K}_{\boldsymbol{linear}}$ into four blocks:

$\boldsymbol{K}_{\boldsymbol{linear}}= \left[ \begin{matrix} \boldsymbol{K}_{train} & \boldsymbol{K}_{cov} \\ \boldsymbol{K}_{cov}^{T} & \boldsymbol{K}_{test} \end{matrix} \right]$,

where $\boldsymbol{K}_{train}$ is the variance matrix of the training set, $\boldsymbol{K}_{test}$ is the variance matrix of the test set, and $\boldsymbol{K}_{cov}$ is the covariance matrix between the training and test sets. We find the maximum likelihood estimator ${\hat{\boldsymbol{\beta}}}_{C}$ for the fixed effects, the best linear unbiased predictor ${\hat{\boldsymbol{U}}}_{train}$ of the omic predictive scored of the training set,and the restricted maximum likelihood estimators $\hat{\sigma}_{m}^{2}$ for the variance components of the fused kernel, using *rrBLUP* [3]. We then estimate the total predictive scores ${\hat{\boldsymbol{Y}}}_{test}$:

$${\hat{\boldsymbol{Y}}}_{test}=\boldsymbol{X}_{C_{test}} {\hat{\boldsymbol{\beta}}}_{C}+\boldsymbol{K}_{cov}\boldsymbol{K}_{train}^{-1}{\hat{\boldsymbol{U}}}_{train}$$

We then compute the adjusted $R^{2}$ between the $\boldsymbol{Y}_{test}$ and ${\hat{\boldsymbol{Y}}}_{test}$ to assess the predictive performance of the omic predictive scores, using Theil’s formula for adjusted $R^{2}$. This process is repeated across all folds and the adjusted $R^{2}$ are averaged to create a predictive index for a given clinical and omic profile. We consider all possible combinations of omics after regressing out clinical covariates in these multiomic kernel models. External validation is conducted similarly; however, as the relevant clinical covariates were not available in the MARBLES dataset, we do not adjust the molecular profiles for these clinical covariates in the predictive models applied to this dataset [4]. In addition, as the MARBLES dataset only contains methylation data, the kernel models considered for external validation only consider the CpG methylation kernel matrix.

***Kernel regularized least squares regression for Gaussian kernels and model evaluation***

We employ a similar Monte Carlo cross-validation scheme, splitting our data into 75%-25% training and test sets across 50 folds. We implement kernel-based regularized squares regression through the *KRLS* package [5], that minimizes the Tikhonov objective function over squared loss. Briefly, the objective of KRLS, in our case, is to find the $c$ that minimizes

$T\left( c \right)= \sum_{i=1}^{n} \left( \boldsymbol{Y}-{\boldsymbol{K}_{\boldsymbol{Gauss}}}_{m}c \right)^{T}(Y-{\boldsymbol{K}_{\boldsymbol{Gauss}}}_{m}c)+\lambda c^{T}{\boldsymbol{K}_{\boldsymbol{Gauss}}}_{m}c$,

where $\lambda$ is tuned via leave-one-out cross-validation. Furthermore, KRLS can compute the pointwise partial derivatives of the fitted function with respect to each predictor using estimators developed by Hainmuller and Hazlett [5]. These pointwise partial derivatives can be used to examine the marginal effect of every feature in the omic design matrix on the outcome of interest.

Using the best parameter estimates from the training set, a Gaussian kernel matrix can be computed from the test set, and we define predicted values in the test set as

${\hat{\boldsymbol{Y}}}_{test}={{\boldsymbol{K}_{\boldsymbol{Gauss}}}_{m}}_{test}\hat{c}$.

We compute adjusted $R^{2}$ between the observed and predicted values of the outcome of interest here, as well, to assess predictive performance.

***Sparse regression model and evaluation***

We also consider linear models to predict outcomes of interest using a regularized regression model:

$\boldsymbol{Y}=\boldsymbol{X}_{\boldsymbol{m}}\boldsymbol{\beta}_{m}+\boldsymbol{\epsilon}$,

where $\boldsymbol{X}_{m}$ and $\boldsymbol{\beta}_{m}$ are the $n\times p_{m}$ design matrix and effects for a given omic profile $m$, using similar Monte Carlo cross-validation to evaluate predictive performance. In the training set, using *glmnet*, we estimate ${\hat{\boldsymbol{\beta}}}_{m}$ using elastic net regularized regression with a mixing parameter of 0.5 (even mixture of LASSO and ridge penalties) and the regularization penalty parameter tuned over 5 folds [6]. Using these parameter estimates, we predict on the test set and evaluate predictive performance using adjusted $R^{2}$.

***Feature selection***

To tune the number of features to include for each omic in the final predictive models, we tuned over various $P$-value thresholds from fold-wise one-way tests of associations. We split the data into 10 even training set-test set folds, conducted one way tests of associations in the training set (i.e. differential expression analysis for mRNA and miRNA expression, EWAS for DNA methylation), and selected all biomarkers with associations with the outcome of interest with $P$-value under a given threshold. Using these biomarkers, we predict the outcome of interest in the test set using both kernel regression methods and compute the adjusted $R^{2}$ to assess predictive performance.

***External validation using MARBLES dataset***

We obtained one external placental CpG methylation dataset from the Markers of Autism Risk in Babies-Learning Early Signs (MARBLES) cohort [4]. To assess out-of-sample performance of kernel models for methylation, we downloaded MethylC-seq data for 47 placenta samples, 24 of which identified as ASD cases (NCBI Gene Expression Omnibus accession numbers GSE67615) [4]. We extracted $\beta$-values for DNA methylation from BED files and transformed into $M$-values with an offset of 1 [7]. We then used the best linear kernel and kernel regression models to predict SRS and IQ in the MARBLES dataset, as detailed above. It is important to note that not all CpG sites used in the best-methylation model from ELGAN were assayed in the MARBLES external validation set (only approximately 85% overlap). Furthermore, the MARBLES dataset does not have measures of SRS or IQ. Thus, to assess the validity of predicted SRS and IQ estimates in MARBLES, we tested for association between the predicted SRS and IQ values and ASD case-control status.

***Reference-based cell-type deconvolution with unmix***

We consider reference-based cell-type deconvolution of bulk placental mRNA expression. Here, from single cell RNA-seq expression profiles for placenta-derived extravillous trophoblasts, cytotrophobalsts, syncytiotrophoblasts, and stromal cells (GSE89497) [8]. We extracted cell-type specific single-cell RNA-seq data and took the median gene expression per gene for these four cell-types. This median expression profile represents an approximate matrix of mRNA expression for a “pure” population of these cells; call this matrix $\boldsymbol{G}$, with 4 columns representing the 4 cell types and 11,338 rows, representing genes that are present in both $\boldsymbol{G}$ and the bulk mRNA expression matrix from ELGAN. Now, using the bulk mRNA expression matrix (call this $\boldsymbol{X}$) from ELGAN, we estimate a matrix of cell-type proportions $\boldsymbol{P}$ using non-negative least squares with numerical optimization done in the variance-stabilized space using unmix from the DESeq2 package [9]. More specifically, the estimated proportion matrix $\hat{\boldsymbol{P}}$ is determined from:

$$\hat{\boldsymbol{P}}={\underset{\boldsymbol{P}}{\mathrm{argmin}} \left\| vst\left( \boldsymbol{X} \right)-vst\left( \boldsymbol{GP} \right) \right\|}_{2},$$

where $vst(\cdot)$ is the variance-stabilizing transformation function from DESeq2 [9]. Carrying out numerical optimization in the variance-stabilized space allows for more efficient estimation with a lower bound.

Reference-based deconvolution has two key advantages: the outputted proportion matrix is easily interpretable and maps back to the cell-type-specific input expression matrix. In this regard, the first column of the estimated proportion matrix $\boldsymbol{P}$ gives the estimated proportions of the cell type represented by the expression profile in the first column of the expression profiles matrix $\boldsymbol{G}$. The use of single-cell RNA-seq inputs derived from placenta tissue also allows for robust characterization of the represented cell-types. However, reference-based deconvolution has a drawback, too. We can only consider cell-types that are represented in the input expression profiles matrix. Hence, we are unable to estimate proportions for endothelial cell populations that comprise bulk placenta tissue but are not captured in the reference single-cell RNA-seq data.

***Differential expression analysis with cell-type proportions***

To detect cell-type-specific differential expression signals, we considered the following model:

$$Y=X_{G}\beta_{G}+\boldsymbol{X}_{\boldsymbol{C}}\beta_{C}+X_{P}\beta_{P}+\left( X_{G}\times X_{P} \right)\beta_{interaction}+\epsilon,$$

where $Y$ is the vector of SRS or IQ outcome, $X_{G}$ is the vector of gene expression for a given gene $G$, $\beta_{G}$ is the effect size of gene expression on the outcome, $\boldsymbol{X}_{\boldsymbol{C}}$ is the matrix of covariates, $\beta_{C}$ is the vector of effect sizes for these covariates, $X_{P}$ is the vector of cell-type proportions for a given cell-type, $\beta_{P}$ is the effect size of cell-type proportion on the outcome, $\beta_{interaction}$ is the effect size of the interaction between gene expression and cell-type proportion, and $\epsilon$ represents random noise. This interaction model subtly changes the interpretation of the main gene expression term $\beta_{G}$, representing an estimate of the gene expression effect size on SRS or IQ at 0% compartment-specific cells. Thus, we recover cell-type-specific differentially expressed genes by testing the interaction effect ($H_{0}: \beta_{interaction}=0$), which measures how the magnitude of the gene-outcome associations differs in bulk tissue with 0% and 100% of the given cell type [10].

**References**

1. Elisseeff A, Technologies B, Shawe-Taylor J, Holloway R, Kandola J. On Kernel-Target Alignment.

2. Zhu B, Song N, Shen R, Arora A, Machiela MJ, Song L, et al. Integrating Clinical and Multiple Omics Data for Prognostic Assessment across Human Cancers. Sci Rep. Nature Publishing Group; 2017;7:16954.

3. Endelman JB. Ridge Regression and Other Kernels for Genomic Selection with R Package rrBLUP. Plant Genome. 2011;4:250–5.

4. Schroeder DI, Schmidt RJ, Crary-Dooley FK, Walker CK, Ozonoff S, Tancredi DJ, et al. Placental methylome analysis from a prospective autism study. Mol Autism. BioMed Central Ltd.; 2016;7:51.

5. Hainmueller J, Hazlett C. Kernel Regularized Least Squares: Reducing Misspecification Bias with a Flexible and Interpretable Machine Learning Approach. Polit Anal. 2014;22:143–68.

6. Friedman J, Hastie T, Tibshirani R. Regularization Paths for Generalized Linear Models via Coordinate Descent. J Stat Softw [Internet]. 2010 [cited 2019 Apr 6];33:1–22. Available from: http://www.jstatsoft.org/v33/i01/

7. Du P, Zhang X, Huang C-C, Jafari N, Kibbe WA, Hou L, et al. Comparison of Beta-value and M-value methods for quantifying methylation levels by microarray analysis. BMC Bioinformatics. 2010;11:587.

8. Liu Y, Fan X, Wang R, Lu X, Dang YL, Wang H, et al. Single-cell RNA-seq reveals the diversity of trophoblast subtypes and patterns of differentiation in the human placenta. Cell Res. Nature Publishing Group; 2018;28:819–32.

9. Love MI, Huber W, Anders S. Moderated estimation of fold change and dispersion for RNA-seq data with DESeq2. Genome Biol. BioMed Central; 2014;15:550.

10. André G;, Westra H-J, Arends D, Esko T, Peters MJ, Schurmann C, et al. Cell Specific eQTL Analysis without Sorting Cells. Cell Specif eQTL Anal without Sorting Cells PLoS Genet. 24:1005223.
