# Supplementary material for: Evidence for the placenta-brain axis: multi-omic kernel aggregation predicts intellectual and social impairment in children born extremely preterm
Source: Mol Autism. 2020 Dec 11;11:97. doi: 10.1186/s13229-020-00402-w (PMC7730750; doi:10.1186/s13229-020-00402-w)
Supplement: Supplementary file 2 — Additional file 2. Supplemental results. [file 13229_2020_402_MOESM2_ESM.docx]

**Supplemental Results**


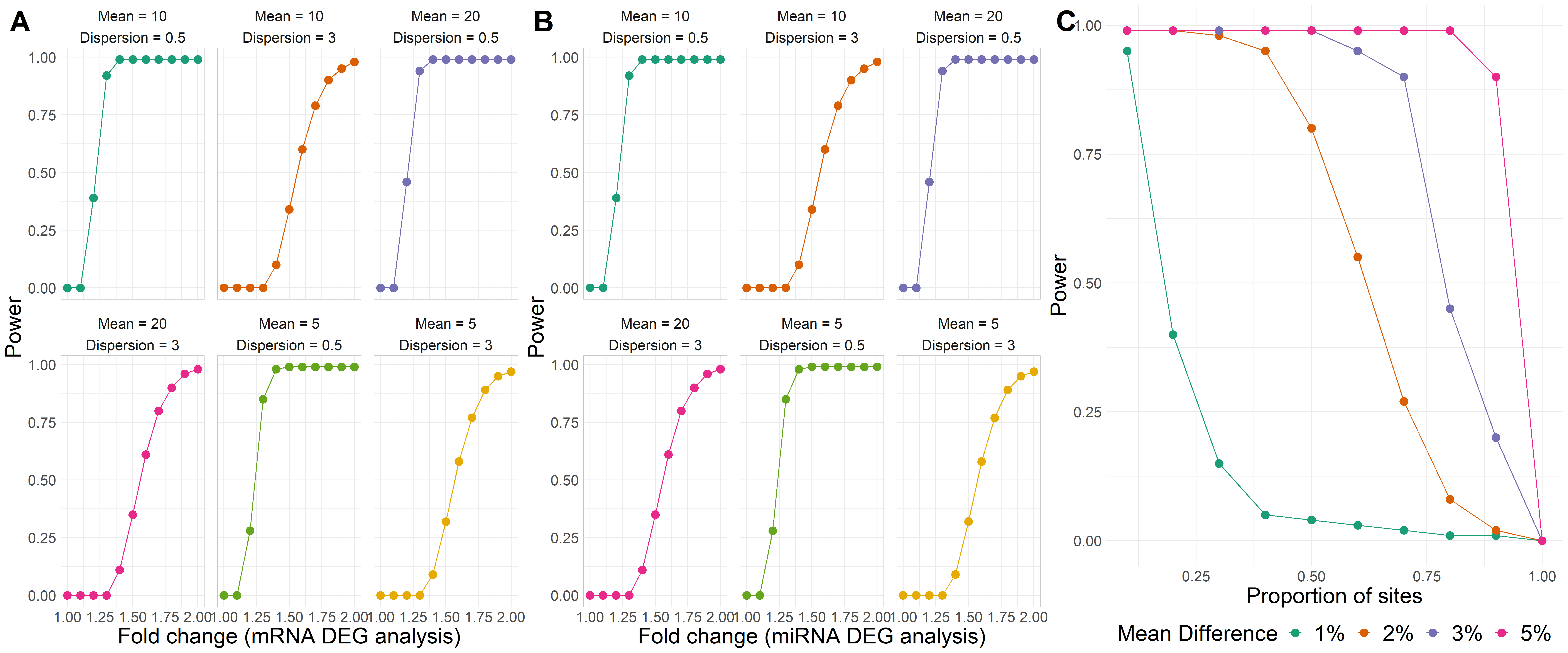


**Figure S1**: Power curves of differential expression and epigenome-wide association analyses. **(A)** For differential mRNA expression analysis, plot of power (Y-axis) to detect a given fold change (X-axis) across different mean and dispersion settings for expression of a gene across 12,000 genes, an FDR of 0.01, a significance level of 0.05, and a sample size of 379. The plot provides the estimated power (Y) to detect the corresponding fold change (X) in a gene whose mRNA expression has provided mean and variance. **(B)** For differential miRNA expression analysis, plot of power (Y-axis) to detect a given fold change (X-axis) across different mean and dispersion settings for a gene across 2,000 genes, an FDR of 0.01, a significance level of 0.05, and a sample size of 379. The plot provides the estimated power (Y) to detect the corresponding fold change (X) in a miRNA whose expression has provided mean and variance. Here the dispersion of a gene measures how much the variance deviates from the mean, as in the traditional definition of a negative binomial distribution. **(C)** For EWAS (differential methylation analysis), plot of power (Y-axis) to detect a given proportion of differentially methylated sites (X-axis) across various effect sizes for 850,000 genes, an FDR of 0.01, a significance level of 0.05, and a sample size of 379. The plot provides the estimated power (Y) to detect a given proportion of sites (X) for a mean difference in 1, 2, 3, or 5% in the outcome per unit of M-value of the CpG site.


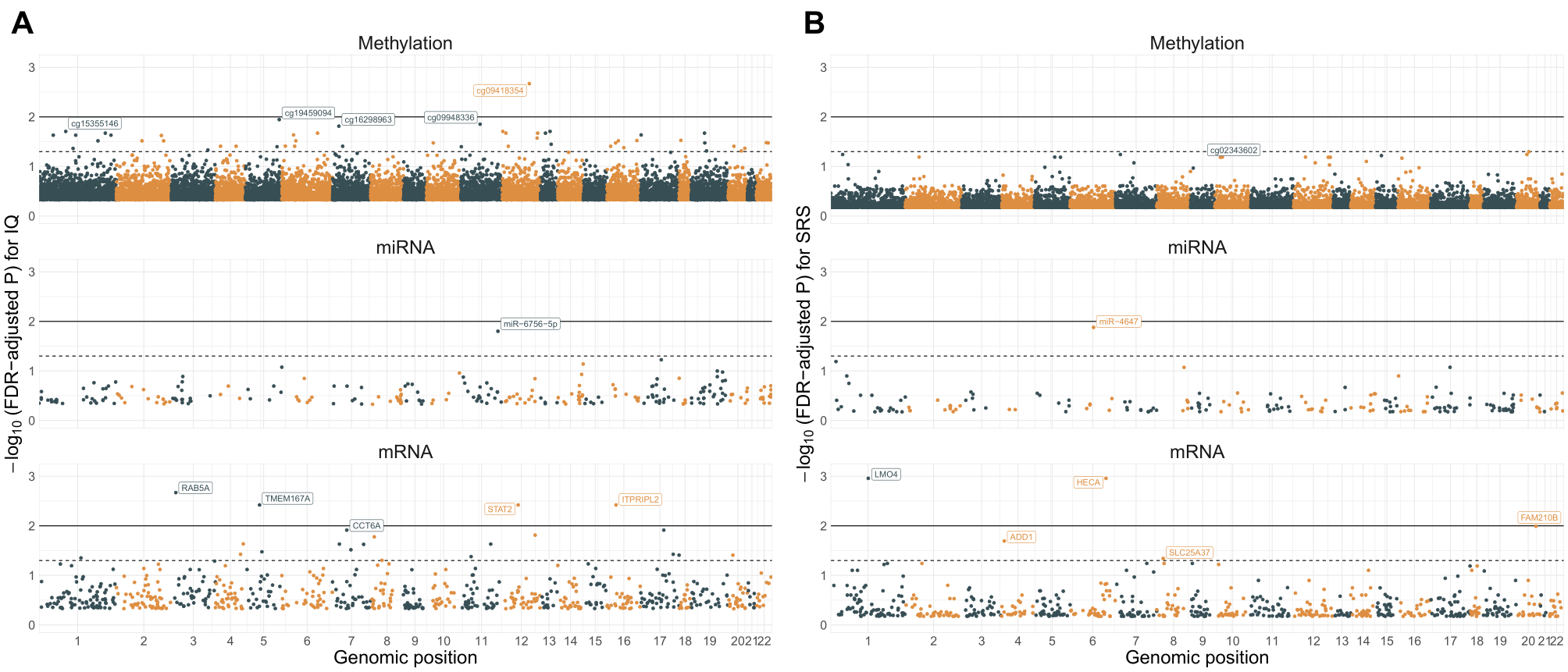


**Figure S2**: Manhattan plots of one-way omic association tests for IQ and SRS. Manhattan plots for one-way tests of association for methylation (top), miRNA (middle), and mRNA expression with IQ **(A)** and SRS **(B)**. The X-axis plots the genomic position of the biomarker and the Y-axis plots the Benjamini-Hochberg FDR-adjusted P-value for the association with the given outcome. The dotted line provides a reference of FDR-adjusted P=0.05, and the solid line provides a reference of FDR-adjusted P=0.01. Biomarkers are labelled with their association has FDR-adjusted P=0.01.


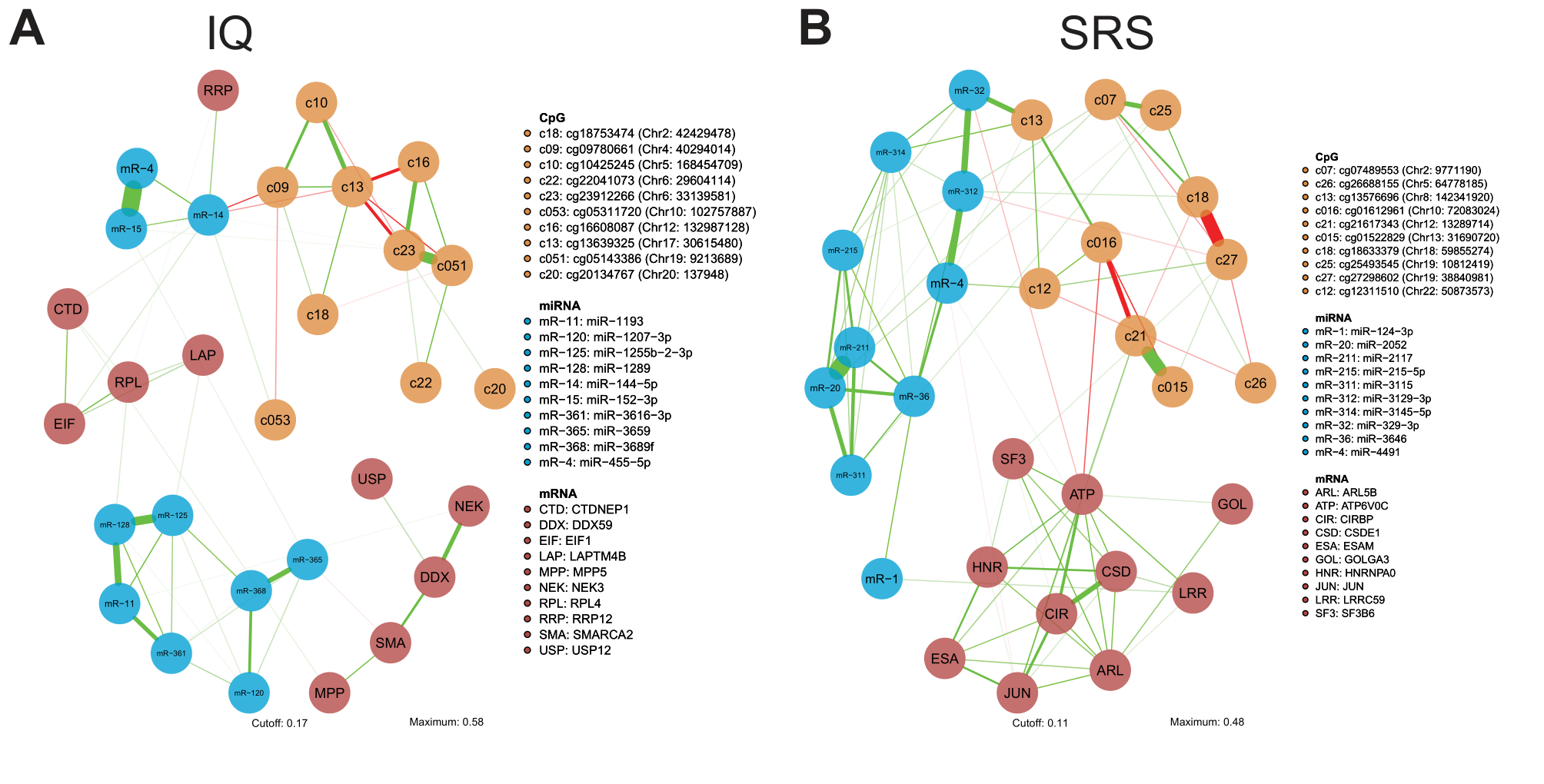


**Figure S3**: Sparse correlative networks between top predictive biomarkers of IQ and SRS. Using the top 50 CpGs (orange nodes), top 50 miRNAs (blue), and top 50 mRNAs (red) that are predictive of IQ (left) and SRS (right), we inferred sparse correlative networks. Nodes are biomarkers and edges show correlations between biomarkers. Positive correlations are shown in green, negative correlations are shown in red, and the thickness of the edge gives the absolute magnitude of correlation.

**Table S1**: Results from association analysis of differentially expression genes with cell-type proportions of the four reference cell types. Regression estimates (beta) and FDR-adjusted P-values (P) from a simple linear model with cell-type proportion as the outcome and log-transformed, normalized gene expression as the primary predictor, adjusted for race, age, sex, number of gestational age days, birth weight $Z$-score, acute inflammation of the placental chorion, and education level of the mother.

| **Gene/CpG** | **Beta, P (Extravillous Trophoblasts)** | **Beta, P (Cytotrophoblasts)** | **Beta, P (Syncytiotrophoblasts)** | **Beta, P (Mesenchymal stromal cells)** |
| --- | --- | --- | --- | --- |
| *HECA* | -0.001, 0.302 | 0.001, 0.176 | -0.002, 0.112 | 0.000, 0.761 |
| *LMO4* | 0.000, 0.813 | 0.000, 0.274 | 0.002, 0.256 | -0.001, 0.504 |
| *RAB5A* | -0.003, 0.181 | 0.000, 0.898 | 0.000, 0.504 | 0.002, 0.176 |
| *TMEM167A* | 0.005, 0.274 | -0.001, 0.184 | 0.000, 0.669 | -0.002, 0.536 |
| *ITPRIPL2* | 0.000, 0.473 | 0.001, 0.234 | 0.000, 0.274 | 0.003, 0.504 |
| *STAT2* | 0.000, 0.373 | 0.000, 0.333 | 0.001, 0.091 | -0.017, 0.091 |
| cg09418354 | -0.002, 0.091 | 0.001, 0.263 | -0.001, 0.184 | 0.016, 0.150 |

**Table S2**: Results from differential mRNA expression analysis of genes with interaction of reference-based estimated proportions of cytotrophoblasts, extravillous trophoblasts, syncytiotrophoblasts, and stromal cells. Effect sizes presented shows the regression parameter estimate for the interaction of gene expression and compartment proportion. Genes presented have at least one differential association with FDR-adjusted P < 0.05.

|  | **Cytotrophoblasts** | | | **Extravillous trophoblasts** | | | **Syncytiotrophoblasts** | | | **Stromal cells** | | |
| --- | --- | --- | --- | --- | --- | --- | --- | --- | --- | --- | --- | --- |
| **Outcome: SRS** | Beta | P | FDR-adjusted P | Beta | P | FDR-adjusted P | Beta | P | FDR-adjusted P | Beta | P | FDR-adjusted P |
| *BRD2* | -0.67 | 0.25 | 1.00 | -0.19 | 0.67 | 0.91 | -1.31 | 0.00 | 0.10 | 1.63 | 0.00 | 0.04 |
| *ZNF618* | -1.54 | 0.01 | 1.00 | 0.42 | 0.41 | 0.79 | -1.49 | 0.00 | 0.10 | 1.86 | 0.00 | 0.04 |
| **Outcome: IQ** |  |  |  |  |  |  |  |  |  |  |  |  |
| *ATP2B1* | 0.52 | 0.44 | 1.00 | -0.24 | 0.71 | 0.98 | 1.94 | 0.00 | 0.03 | -1.58 | 0.00 | 0.21 |
| *FAM126A* | 0.80 | 0.26 | 1.00 | -1.83 | 0.01 | 0.66 | 2.02 | 0.00 | 0.03 | -1.34 | 0.00 | 0.66 |

**Table S3**: Enriched biological process ontologies for genes identified in correlative networks predictive of IQ and SRS. We provide the functional category of the biological process enriched by this set of genes, the FDR-adjusted P-value of the enrichment, the number of genes out of the inputted set of genes that are found in each gene ontology, and the total number of genes across the protein-coding transcriptome in the ontology.

| **Functional category** | **FDR-adjusted P-value** | **Number of inputted genes in ontology** | **Total number of genes in ontology** |
| --- | --- | --- | --- |
| *IQ* |  |  |  |
| Endomembrane system organization | 0.014 | 3 | 204 |
| Membrane organization | 0.020 | 3 | 296 |
| *SRS* |  |  |  |
| Nucleic acid binding | 0.011 | 5 | 2056 |
| RNA binding | 0.011 | 3 | 536 |
| Ubiquitin protein ligase binding | 0.011 | 2 | 181 |
| Ubiquitin-like protein ligase binding | 0.011 | 2 | 192 |
| Organic cyclic compound binding | 0.014 | 6 | 3510 |
| Heterocyclic compound binding | 0.014 | 6 | 3472 |
| Enzyme binding | 0.039 | 3 | 1230 |
